# MIMRDA: A Method Incorporating the miRNA and mRNA Expression Profiles for Predicting miRNA-Disease Associations to Identify Key miRNAs (microRNAs)

**DOI:** 10.3389/fgene.2022.825318

**Published:** 2022-01-27

**Authors:** Xianbin Li, Hannan Ai, Bizhou Li, Chaohui Zhang, Fanmei Meng, Yuncan Ai

**Affiliations:** ^1^ State Key Laboratory for Biocontrol, School of Life Sciences, Sun Yat-sen University, Guangzhou, China; ^2^ Department of Electrical and Computer Engineering, The Grainger College of Engineering, University of Illinois at Urbana-Champaign, Urbana, IL, United States; ^3^ National Center for Quality Supervision and Inspection of Automatic Equipment, National Center for Testing and Evaluation of Robots (Guangzhou), CRAT, SINOMACH-IT, Guangzhou, China

**Keywords:** MIMRDA prediction method, microRNA (or miRNA), miRNA-disease association, survival analysis, drug resistance, drug sensitivity

## Abstract

Identifying cancer-related miRNAs (or microRNAs) that precisely target mRNAs is important for diagnosis and treatment of cancer. Creating novel methods to identify candidate miRNAs becomes an imminent Frontier of researches in the field. One major obstacle lies in the integration of the state-of-the-art databases. Here, we introduce a novel method, MIMRDA, which incorporates the **mi**RNA and **m**RNA expression profiles for predicting mi**R**NA-**d**isease **a**ssociations to identify key miRNAs. As a proof-of-principle study, we use the MIMRDA method to analyze TCGA datasets of 20 types (BLCA, BRCA, CESE, CHOL, COAD, ESCA, HNSC, KICH, KIRC, KIRP, LIHC, LUAD, LUSC, PAAD, PRAD, READ, SKCM, STAD, THCA and UCEC) of cancer, which identified hundreds of top-ranked miRNAs. Some (as Category 1) of them are endorsed by public databases including TCGA, miRTarBase, miR2Disease, HMDD, MISIM, ncDR and mTD; others (as Category 2) are supported by literature evidences. miR-21 (representing Category 1) and miR-1258 (representing Category 2) display the excellent characteristics of biomarkers in multi-dimensional assessments focusing on the function similarity analysis, overall survival analysis, and anti-cancer drugs’ sensitivity or resistance analysis. We compare the performance of the MIMRDA method over the Limma and SPIA packages, and estimate the accuracy of the MIMRDA method in classifying top-ranked miRNAs via the Random Forest simulation test. Our results indicate the superiority and effectiveness of the MIMRDA method, and recommend some top-ranked key miRNAs be potential biomarkers that warrant experimental validations.

## Introduction

Cancer-related microRNAs (miRNAs) targeting mRNAs affect cell differentiation, proliferation, migration and apoptosis, leading to initiation or prevention of cancer (Evan and Vousden 2001; Bartel 2004; Esquela-Kerscher and Slack 2006). Identifying cancer-related miRNAs to be biomarkers roots in the promising diagnosis and treatment of cancer (Rupaimoole and Slack, 2017; Chen et al., 2019; Zhao, Chen, and Yin 2019). Methods and databases have been developed over decades, including but not limited to miRGen (Megraw et al., 2007), miR2Disease (Jiang, et al., 2009), MiRCancer (Xie et al., 2013), HMDD (Li L. et al., 2014), HMDD 3.0 (Huang, et al., 2019), miRWalk (Dweep and Gretz 2015), dbDEMC (Yang et al., 2017), ncDR (Dai et al., 2017), mTD (Chen et al., 2017a), MISIM (Li et al., 2019), miRbase (Kozomara et al., 2019), DBMDA (Zheng et al., 2020) and miRTarBase (Huang et al., 2020). Creating novel methods to identify candidate miRNAs has become an imminent Frontier of researches in the field.

There are two approaches: the complex network-based methods and the machine learning-based methods (Chen, et al., 2019). The former approach relies on the complex network that integrated miRNA similarity network, disease similarity network and known miRNA-disease relationship network to predict miRNA-disease connections (Jiang et al., 2010). This family includes WBSMDA (Chen et al., 2016a), RWRMDA (Chen et al., 2012), HGIMDA (Chen et al., 2016b) and PBMDA (You et al., 2017). These methods constructed local networks of the miRNA and disease similarity to infer global networks; but the prediction with limited information is of poor quality. The hypergeometric distribution or binomial distribution was fundamentally assumed in most methods, similar to that of the Limma package (Ritchie et al., 2015) and the SPIA package (Tarca et al., 2009). The latter approach applies machine learning (supervised or semi-supervised) techniques to predict miRNA-disease connections. Some examples are the SVM classifier (Xu et al., 2011), HDMP (Xuan et al., 2013), RLSMDA (Chen and Yan 2014), RBMMDA (Chen et al., 2015), MCMDA (Li C. et al., 2017) and RKNNMDA (Chen et al., 2017b). These methods performed better in some cases. Yet, the need for fine-tuning parameters inevitably hinders applications in complex biological systems.

Three works pioneered a new direction through incorporating the miRNA and mRNA expression profiles. One was to construct a relationship network between miRNAs and their target mRNAs (disease-genes) by utilizing the limited miRNA and mRNA expression profiles (Xu et al., 2014). Another was to construct a subnetwork between the disease similarity and the miRNA similarity derived from multiple data-sources (Liu et al., 2017). The third was to construct an mRNA-miRNA-lncRNA network prognostic for triple-negative breast cancer (Huang et al., 2021). However, problems remain challenging due to insufficient relationships between miRNAs and mRNAs (disease-genes) in databases.

The major gap in the field is how to integrate sophisticated databases to identify key miRNAs associated with diseases. This article introduces a novel method, MIMRDA, which incorporates the **mi** RNA and **m** RNA expression profiles for predicting mi **R** NA-**d** isease **a** ssociations to identify key miRNAs. As a proof-of-principle study, we use the MIMRDA method to analyze TCGA datasets of 20 types of cancer (comprising 10,449 samples), followed by functional cross-verification through utilizing multiple sophisticated databases including miR2Disease (Jiang, et al., 2009), HMDD 3.0 (Huang, et al., 2019), ncDR (Dai et al., 2017), mTD (Chen et al., 2017a), MISIM 2.0 (Li et al., 2019) and miRTarBase (Huang et al., 2020). We evaluate the superiority of the MIMRDA method to the Limma and SPIA packages (Tarca et al., 2009; Ritchie et al., 2015). We estimate the accuracy of the MIMRDA method in classifying top-ranked miRNAs via the Random Forest simulation test. We discuss some top-ranked key miRNAs with experimental evidences drawn from literature, suggesting their potential to be biomarkers for clinical applications.

## Materials and Methods

### Design and Implementation of the MIMRDA Method

**FIGURE 1:**
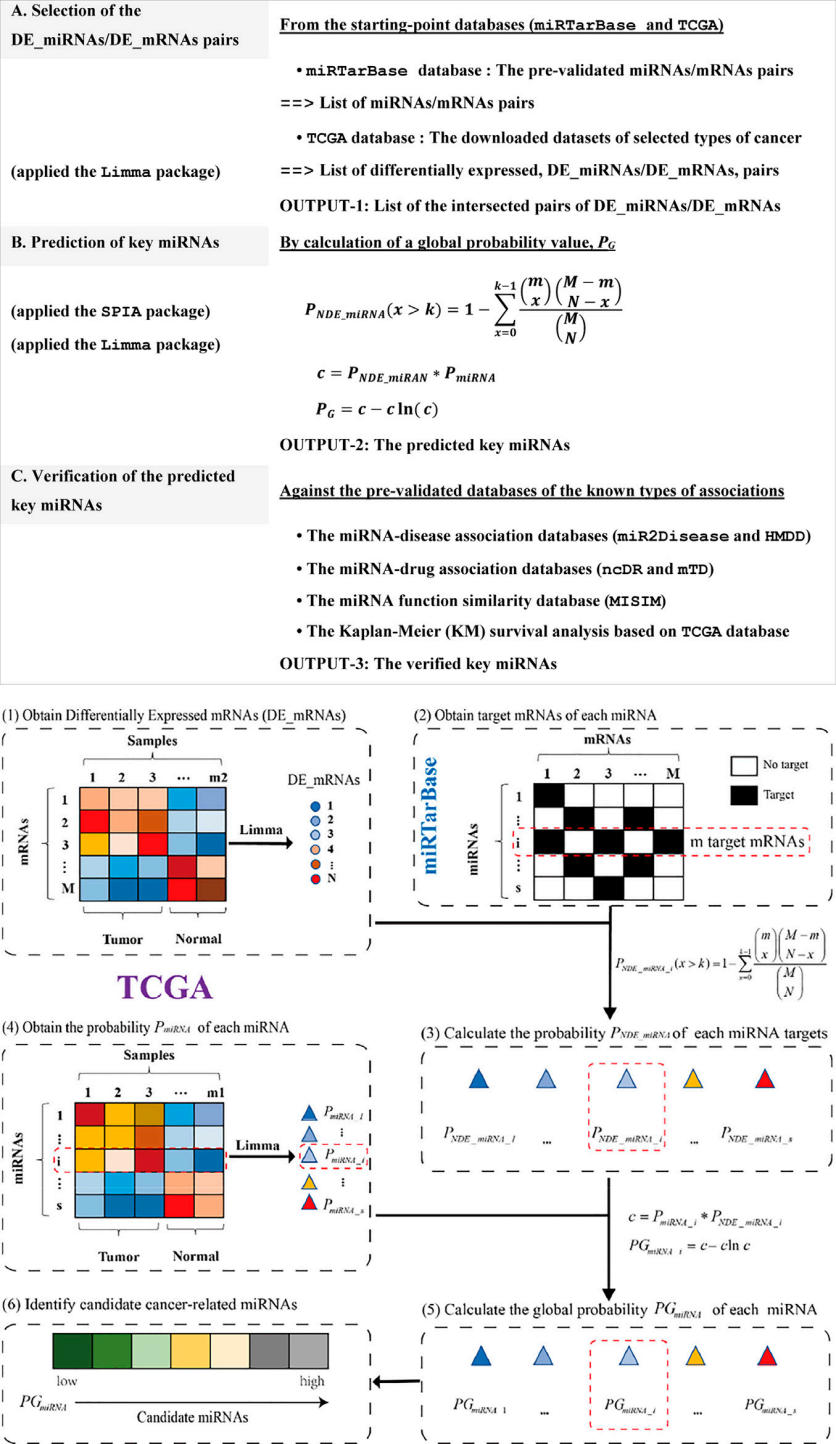
Workflow of the MIMRDA method. Multiple steps for predicting and verifying key miRNAs (top-box). Sequential procedures for calculating a global probability *P*_*G*_ value (bottom-box). The probability *P*_*miRNA*_ is estimated by using the Limma package for DE_miRNAs from a TCGA dataset. The probability *P*_*NDE_miRNA*_ is estimated with the formula, which incorporates the expression profiles of miRNAs and their target mRNAs from both TCGA dataset and miRTarBase database. The global probability *P*_*G*_ is adjusted by the Fisher’s product of *P*_*NDE_miRNA*_ and *P*_*miRNA*_. Symbols: Total number of DE_mRNAs (***N***) present in a given TCGA dataset; Total number of mRNAs (***M***) and the number of DE_mRNAs (***k***), as well as the number of mRNAs (***m***) that are precisely targeted by the *i*th miRNA (*i* being the current step in the iteration) present in the miRTarBase database. See the main text for details.

The miRNA-disease association prediction method (MIMRDA) incorporated the expression profiles of both miRNAs and mRNAs to identify key miRNAs. The demo R code was freely available at https://github.com/eshinesimida/MIMRDA. The datasets from TCGA (https://portal.gdc.cancer.gov/) and miRTarBase (Huang et al., 2020) were used as starting-points, followed by multiple steps for predicting and verifying the key miRNAs that were significantly related to at least one type of cancer (Figure 1, top-box). Key miRNAs were predicted at the significance level of global probability *P*_*G*_, for which the Differentially Expressed miRNAs (DE_miRNAs) and their target mRNAs (DE_mRNAs) were essentially measured (Figure 1, bottom-box). The sequential procedures were outlined below.

**Firstly**, we counted the total number of DE_mRNAs (***N***) that were identified from a TCGA dataset by using the Limma package (Ritchie et al., 2015) at the significance level of BH-adj. Pval <0.01. **Secondly**, we estimated the probability *P*_*miRNA*_ based on DE_miRNAs in the TCGA dataset by using the Limma package (Ritchie et al., 2015) at the significance level of BH-adj. Pval <0.01. **Thirdly**, we extracted the miRNAs and their target mRNAs, whose associations had been experimentally pre-validated in the miRTarBase database (Chou et al., 2018; Huang et al., 2020), while counting the total number of mRNAs (***M***) and the total number of DE_mRNAs (***k***), as well as the number of DE_mRNAs (***m***) that were precisely targeted by the *i*th miRNA (*i* being the current step in the iteration) from the miRTarBase database. **Fourthly**, we estimated the probability *P*_*NDE_miRNA*_ with an over-representation analysis (ORA) via the SPIA package (Tarca et al., 2009), assuming that the number of DE_miRNAs (that precisely targeted DE_mRNAs) followed a hypergeometric distribution with three parameters (***N***, ***M*** and ***k***). These parameters included the total number of DE_mRNAs (***N***) observed in a given TCGA dataset, the total number of mRNAs (***M***) plus the number of DE_mRNAs (***k***) observed in the miRTarBase database, and the number of mRNAs (***m***) that were precisely targeted by the *i*th miRNA (*i* being the current step in the iteration) observed in the miRTarBase database. Statistically, the probability *P*_*NDE_miRNA*_ value represented the probability of observing the DE_miRNAs for a given number of times or higher, just by chance. **Finally**, we generated the global probability (*P*_*G*_) by adjusting the Fisher’s product of *P*_*NDE_miRNA*_ and *P*_*miRNA*_. The global probability *P*_*G*_ value was used not only to rank DE_miRNAs, but also to choose a desired level of type I error. Small *P*_*G*_ values could occur by chance when multiple testing were simultaneously performed. The FDR-adjusted *P*_*G*_ value was used for controlling the false discovery rate (FDR).

### Case Studies: Evaluating the MIMRDA Method

As a proof-of-principle study, we employed the MIMRDA method to analyze TCGA datasets of 20 types of cancer, comprising 10,499 samples (Table 1). The miRNAs and mRNAs expression profiles along with clinical information were downloaded at the TCGA data portal (https://portal.gdc.cancer.gov/) (as of April 30, 2020). The Limma package (Ritchie et al., 2015) was deployed to extract differentially expressed mRNAs (DE_mRNAs) and miRNAs (DE_miRNAs), respectively, from each dataset. The Benjamini–Hochberg adjusted *p*-value (BH-adj.*p*-value) < 0.01 was used to select significantly, differentially expressed entities (DE_mRNAs and DE_miRNAs).

**TABLE 1:** Datasets of 20 types of cancer downloaded from TCGA.

Cancer	Total	Tumor	Normal	Details
BLCA	453	416	37	Bladder Urothelial Carcinoma
BRCA	1,282	1,120	162	Breast invasive carcinoma
CESE	317	309	8	Cervical squamous cell carcinoma
CHOL	71	51	20	Cholangiocarcinoma
COAD	570	477	93	Colon adenocarcinoma
ESCA	251	186	65	Esophageal carcinoma
HNSC	612	530	82	Head and Neck squamous cell carcinoma
KICH	190	113	71	Kidney Chromophobe
KIRC	985	543	442	Kidney renal clear cell carcinoma
KIRP	380	292	88	Kidney renal papillary cell carcinoma
LIHC	469	380	89	Liver hepatocellular carcinoma
LUAD	877	603	274	Lung adenocarcinoma
LUSC	765	511	254	Lung squamous cell carcinoma
PAAD	221	185	36	Pancreatic adenocarcinoma
PRAD	623	505	118	Prostate adenocarcinoma
READ	192	173	19	Rectum adenocarcinoma
SKCM	477	474	3	Skin Cutaneous Melanoma
STAD	544	443	101	Stomach adenocarcinoma
THCA	615	515	100	Thyroid carcinoma
UCEC	605	553	52	Uterine Corpus Endometrial Carcinoma

### Cross-Verification of key miRNAs Against the miRNA-Disease Association Databases (miR2Disease and HMDD)

The miR2Disease database (http://www.miR2Disease.org) was manually curated, containing miRNAs related to human diseases (Jiang, et al., 2009). Each entry contained information about the miRNA-disease association, including miRNA ID, disease name, brief description of the relationship, miRNA expression pattern, miRNA expression detection method, target genes that were experimentally pre-verified in literature. This database currently comprised 3,273 entries, involving 349 miRNAs related to 163 human diseases (as of April 30, 2021). The HMDD 3.0 database (Huang, et al., 2019) currently contained 5,430 types of relationship between 495 miRNAs and 383 diseases (as of April 30, 2021), which was employed to infer the miRNA-disease associations. The miRNA-disease pairs were downloaded (as of April 30, 2021) at http://www.cuilab.cn/hmdd for analysis.

### Cross-Verification of key miRNAs Against the Function Similarity Database (MISIM)

The MISIM 2.0 database (http://www.lirmed.com/misim/) (Li et al., 2019) integrated the co-expression similarity, GO function similarity and disease similarity. It was applied to manifest the functional similarity of miRNAs as a tool for the miRNA function analysis (Wang et al., 2010). We deployed the known miRNA-disease interactions to evaluate the functional similarity of miRNAs because miRNAs with similar functions should tentatively associate with similar diseases (Chen D. et al., 2018; Che et al., 2019; Zheng et al., 2020).

### Cross-Verification of key miRNAs via the Kaplan-Meier (KM) Survival Analysis Based on TCGA Database

The Kaplan-Meier (KM) method (Saluja et al., 2019) was used to evaluate the prognostic survival rate of key miRNAs. The median values of miRNAs expression were calculated. miRNAs with expression values higher than the median value were considered to be highly expressed, and vice versa. The TCGA database (with clinical information of patients) was employed to screen the significantly, differentially expressed miRNAs (DE_miRNAs) and determine whether such miRNAs were related to the overall survival (OS). The hazard ratio (HR) and *p*-value were estimated to evaluate the direct relationship between miRNA and prognostic survival. A *p*-value < 0.05 was considered statistically significant.

### Cross-Verification of key miRNAs Against the miRNA-Drug Association Databases (ncDR and mTD)

An miRNA targeting mRNAs caused sensitivity or resistance to anti-cancer drugs. We applied top-20 ranked miRNAs to search against two databases, ncDR (Dai et al., 2017) and mTD (Chen et al., 2017b), looking for candidate matches, thus predicted possible resistance or sensitivity to anti-cancer drugs. These two databases currently contained 5,661 and 3,669 miRNAs-drugs interactions for all diseases (as of October 2021), respectively, which provided information about the dysfunctions of non-coding RNAs (ncRNAs), leading to resistance or sensitivity to anti-cancer drugs.

### Comparison on the Performance of the MIMRDA Method Over Existing Methods

No similar methods was available for side-by-side comparisons. We compared the number distribution of top-ranked miRNAs identified by the MIMRDA method (*P*_*G*_), the Limma package (*P*_*miRNA*_) and the SPIA package (*P*_*NDE_miRNA*_), respectively, at the significance level of adj. Pval <0.01 since the MIMRDA method rooted in the usage of the Limma package (Ritchie et al., 2015) and the SPIA package (Tarca et al., 2009) (see Figure 1). For simplicity, we focused on comparing the number distribution of top-100 ranked miRNAs obtained by these three methods from each dataset of each type of cancer. The more the identified disease-related miRNAs were flagged, the better the method performed.

### Evaluating the Performance of MIMRDA via the Random Forest Simulation Test

To evaluate the accuracy of the MIMRDA method in classifying top-ranked miRNAs, we employed a machine learning method, i.e., the five-fold cross-validation Random Forest (RF), for simulation test (Speiser et al., 2019). Samples of each dataset from each type of cancer were divided (at a ratio of 4:1) into the training and testing sets, respectively. The five-fold cross-validation RF simulation generated a predicted value. We obtained an AUC value by comparing the predicted value with an actual value, and thus compared the MIMRDA method top-ranked (top_5, top_10, top_15, top_20) miRNAs with the randomly selected (random_5, random_10, random_15, random_20) miRNAs, both after the RF simulations. These processes were repeated 1,000 times in order to get a set of AUC values. We then used the AUC-based statistics analysis to evaluate the accuracy of the MIMRDA method in classifying the top-ranked miRNAs. The larger the AUC value was, the better the accuracy of the method classified. The difference was considered statistically significant at *p*-value < 0.001.

## Results

### Identification of miRNAs and Their Target mRNAs

**FIGURE 2:**
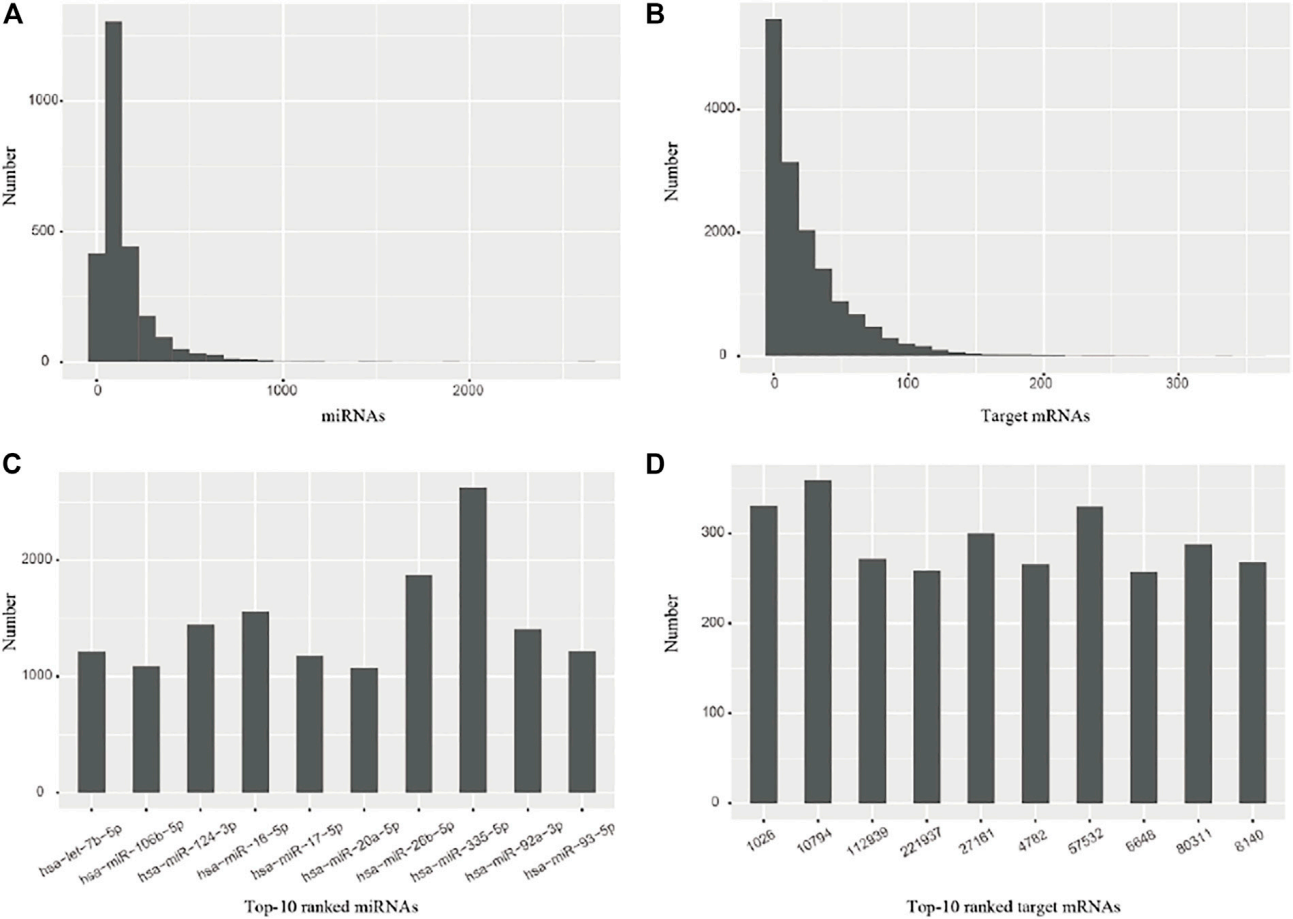
The number distribution of miRNAs and their target mRNAs. **(A)** miRNAs **(B)** Target mRNAs **(C)** Top-10 ranked miRNAs. **(D)** Top-10 ranked target mRNAs.

The miRNAs and their target mRNAs were extracted from the miRTarBase database (Huang et al., 2020) with the experimentally pre-validated miRNA-target associations. The number distribution of miRNAs and mRNAs, respectively, indicates that the majority of miRNAs have 200–300 target mRNAs (Figure 2A), while the majority of target mRNAs have 20–50 miRNAs (Figure 2B); Top-10 ranked miRNAs have more than 1,000 target mRNAs (Figure 2C), while top-10 ranked target mRNAs have more than 250 miRNAs (Figure 2D). These data suggest that such diverse samples are appropriate for subsequent analysis.

### Identification of the Differentially Expressed miRNAs and mRNAs

**FIGURE 3:**
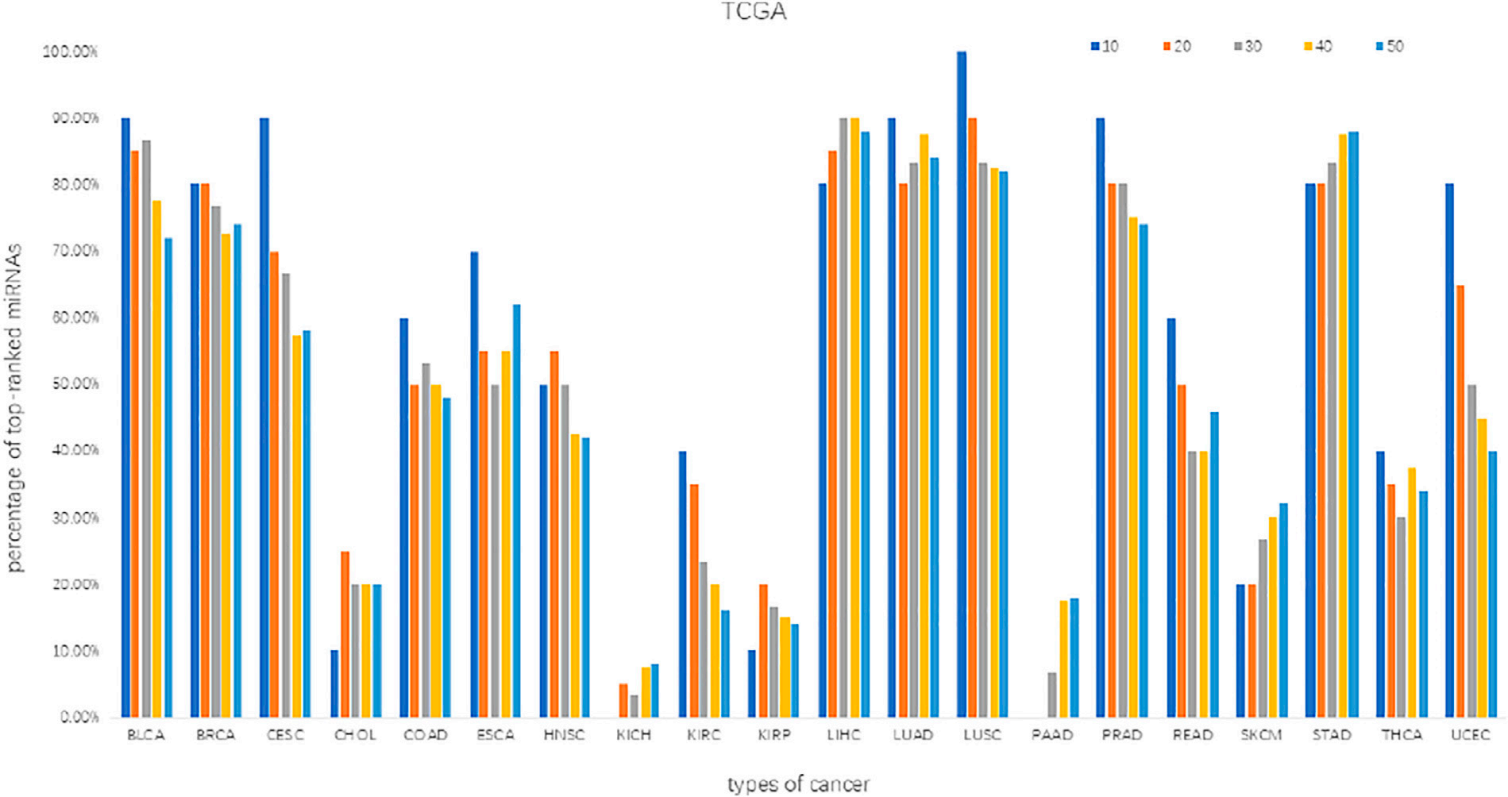
The percentage distribution of top-ranked miRNAs screened from the datasets of 20 types of cancer.

We screened the differentially expressed miRNAs (DE_miRNAs) and target mRNAs (DE_mRNAs) from each dataset by using the Limma package (Ritchie et al., 2015) at the significance level of BH-adj. Pval <0.01. The percentage distribution of top-ranked (top-10, 20, 30, 40, 50) miRNAs indicates that most miRNAs are significantly essential in biology (Figure 3). Note that the percentage of top-ranked miRNAs is a proportion of the top-ranked miRNAs out of the total cancer-related miRNAs that were identified from the given datasets of a cancer type. For instance, surveyed against the HMDD database, we obtained the top-10 ranked miRNAs from the BLCA datasets, of which only nine miRNAs were identified to be truly associated with BLCA, thus yielding a percentage of 90%. The percentage distribution of such top-50 ranked miRNAs suggests an accuracy greater than 70% in BLCA, BRCA, LIHC, LUAD, LUSC, PRAD and STAD datasets, and an accuracy less than 40% in CHOL, KICH, KIRP, PAAD, SKCM and THCA datasets. Similar surveys with the top-10 ranked miRNAs suggest an accuracy greater than 60% in the majority of datasets. These data indicate the effectiveness of the MIMRDA method in identifying key miRNAs that were significantly, differentially expressed in the datasets from 20 types of known cancer, suggesting that they are closely related to the 20 types of known cancer (see Table 1).

### The Impacts of key miRNAs on Multiple Types of Cancer

**FIGURE 4:**
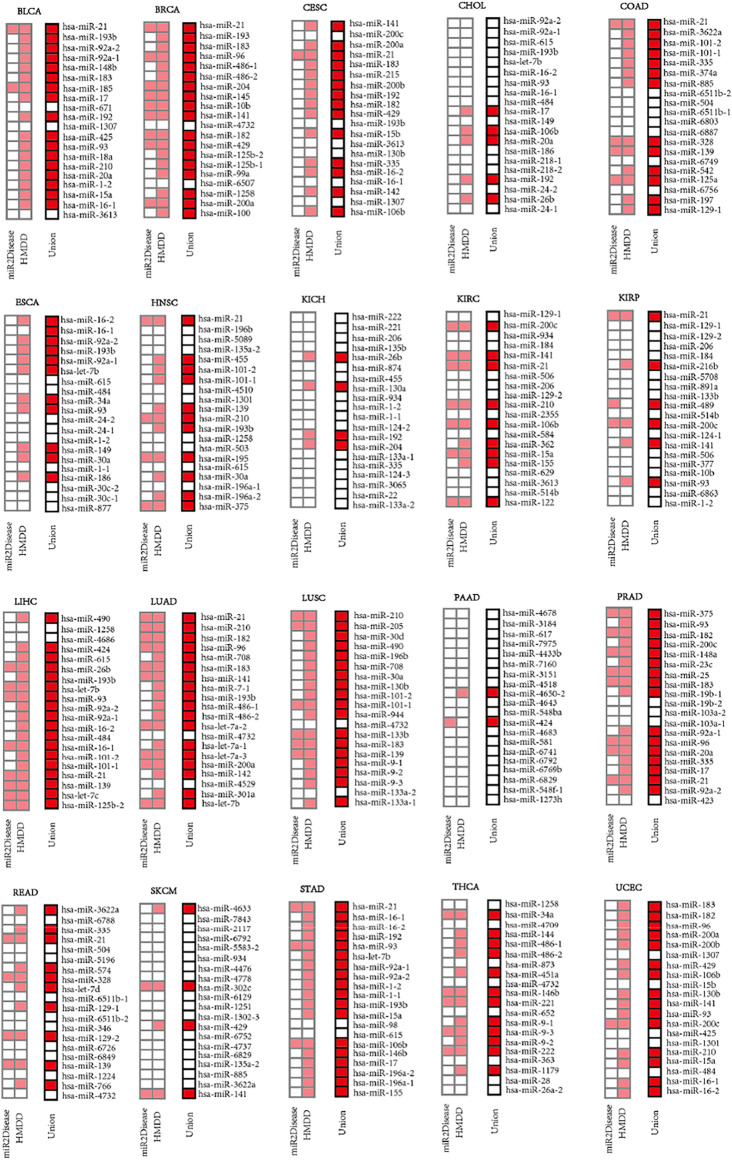
Top-20 ranked miRNAs on the lists of priorities (squares in light red or red color) for 20 types of cancer when searched against the miR2Disease and HMDD databases, respectively.

We extracted top-20 ranked miRNAs from each dataset and searched them against the miRNA-disease association databases (miR2Disease and HMDD) whose biological functions had been pre-verified clinically or experimentally. The results (Figure 4) indicated that more than 50% of the top-20 ranked miRNAs were related to 14 types of cancer (BLCA, BRCA, CESC, COAD, ESCA, HNSC, LIHC, LUAD, LUSC, PRAD, STAD, THCA and UCEC), despite that certain top-20 ranked miRNAs were not related to any cancer type at all. We identified perfect matches (defined as Category 1), including 1) 18 miRNAs were from BRCA, LIHC, LUAD, LUSC and STAD; 2) 17, 16, 15, 14, 13, 12, 11, 11, 10 miRNAs separately were from BLCA, PRAD, UCEC, CESC, COAD, THCA, ESCA, HNSC, READ; and 3) less than 10 miRNAs were from CHOL, KICH, KIRC, KIRP, PAAD and SKCM. Strikingly, the MIMRDA method suggested that certain top-20 ranked miRNAs (e.g., **miR-1258** and **miR-4686**) were related to cancer, but they were beyond (i.e., they were not matched with) the current version of miR2Disease and HMDD databases. We defined these candidate miRNAs as Category 2, which warrant validations in future experiments.

Among the 198 miRNAs out of the top-20 ranked miRNAs screened from the 20 types of cancer, 85 miRNAs were related to multiple types of cancer whereas the rest 113 miRNAs were related to one cancer type (Figure 4). Those key miRNAs related to multiple types of cancer will be discussed (in Discussion) later with accumulated experimental evidences drawn from literature. Here, we highlight certain cases that were related to single type of cancer. 1) Four (miR-148b, miR-185, miR-671 and miR-18a) were related to BLCA, and ranked 5th, 7th, 9th and 14th, respectively. 2) Five (miR-145, miR-125b01, miR-99a, miR-6507 and miR-100) were related to BRCA, and ranked 8th, 15th, 16th, 17th and 20th, respectively. 3) One (miR-215) was related to CESC, and ranked 6th. 4) Two (miR-218-1 and miR-218-2) ranked 15th and 16th were related to CHOL. 5) Eight (miR-74a, miR-6803, miR-6887, miR-6749, miR-542, miR-125a, miR-6756 and miR-197) were related to COAD, and ranked 6th, 11th, 12th, 15th, 16th, 17th, 18th and 19th, respectively. 6) Three (miR-30c-2, miR-30c-1 and miR-877) were related to ESCA, and ranked 18th, 19th and 20th, respectively. 7) Four (miR-5089, miR-4510, miR-503 and miR-195) were related to HNSC, and ranked 3rd, 8th, 14th and 15th, respectively. 8) Seven (miR-135b, miR-874, miR-130a, miR-124-2, miR-124-3, miR-3065 and miR-22) were related to KICH, and ranked 4th, 6th, 8th, 12th, 17th, 18th and 19th, respectively. 9) Five (miR-2355, miR-584, miR-362, miR-629 and miR-20) were related to KIRC, and ranked 11th, 13th, 14th, 17th and 20th, respectively. 10) Seven (miR-216b, miR-4508, miR-891a, miR-489, miR-124-1, miR-377 and miR-6863) were related to KIRP, and ranked 6th, 7th, 8th, 10th, 13th, 16th and 19th, respectively. 11) Two (miR-4686 and let-7c) were related to LIHC, and ranked 3rd and 9th, respectively. (**xii**) Six (miR-7-1, let-7a-2, let-7a-1, let-7a-3, miR-4529 and miR-310a) were related to LUAD, and ranked 8th, 12th, 14th, 15th, 18th and 19th, respectively. (**xiii**) Three (miR-205, miR-30d and miR-944) were related to LUSC, and ranked 2nd, 3rd and 11th, respectively. (**xiv**) Nine (miR-6788, miR-5196, miR-574, let-7d, miR-346, miR-6726, miR-6849, miR-1224 and miR-766) were related to READ, and ranked 2nd, 6th, 7th, 9th, 13th, 15th, 16th, 18th and 19th. (**xv**) One (miR-98) was related to STAD, and ranked 13th. (**xvi**) Remarkably, no miRNAs was related to UCEC at all. Taken together, these data suggest that the MIMRDA method is effective in identifying key miRNAs from specific type of cancer.

### Verification of key miRNAs via the Biological Function Similarity Analysis

**FIGURE 5:**
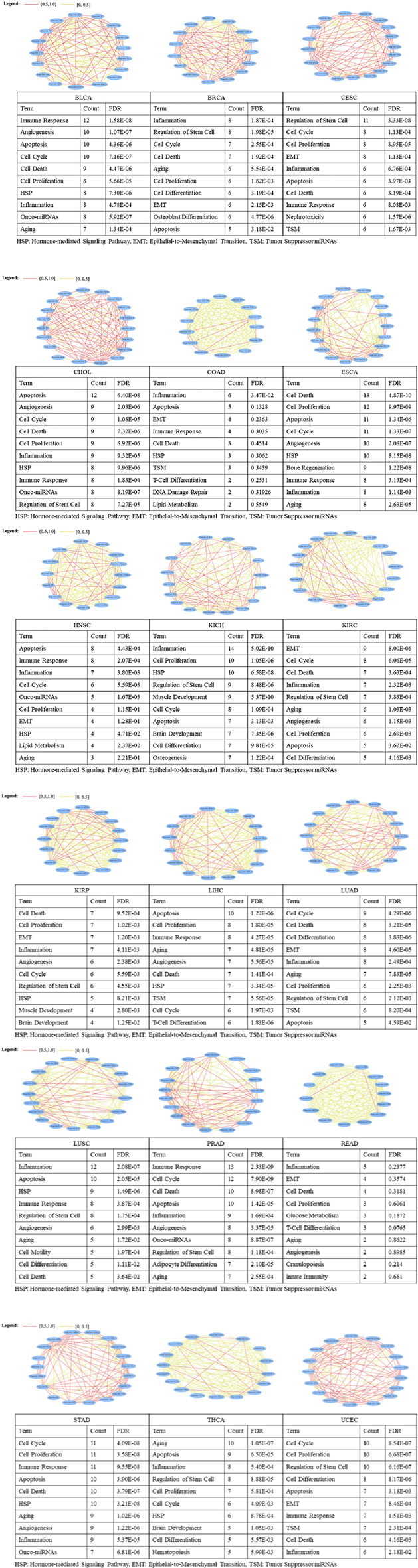
Biological function similarity analysis of the top-20 ranked miRNAs.

We applied MISIM 2.0 database to annotate the top-20 ranked miRNAs from each dataset of the 20 types of cancer (Figure 5). The findings revealed that the majority of top-20 ranked miRNAs were annotated, including 19 in CHOL and STAD; 18 in CESC, ESCA, KIRC, LUSC and PRAD; 17 in BLCA, KICH, THCA and UCEC; 16 in KIRP, LIHC and LUAD; 15 in BRCA and HNSC; and 14 in COAD and READ. However, none of the top-20 ranked miRNAs was annotated in PAAD and SKCM. Meanwhile, the function similarity network of the top-20 ranked miRNAs indicated that the majority of miRNAs were highly related to one another in biological functions, as the red line represents that the correlation coefficient is greater than 0.5 (Figure 5). For instance, the top-10 ranked miRNAs are corresponding to the enriched biological functions (FDR <0.05), which are mainly involved in cell cycle, proliferation, inflammation, death and apoptosis (Figure 5). And these functions have been experimentally pre-verified to be closely associated with various types of cancer (Evan and Vousden 2001; Taniguchi and Karin 2018). These results suggest that such key miRNAs possess highly coupled linkages, which drive the essential biological functions at the system-level, thereby enhancing their potential of clinical applications.

### Verification of key miRNAs via the Kaplan-Meier (KM) Survival Analysis

**FIGURE 6:**
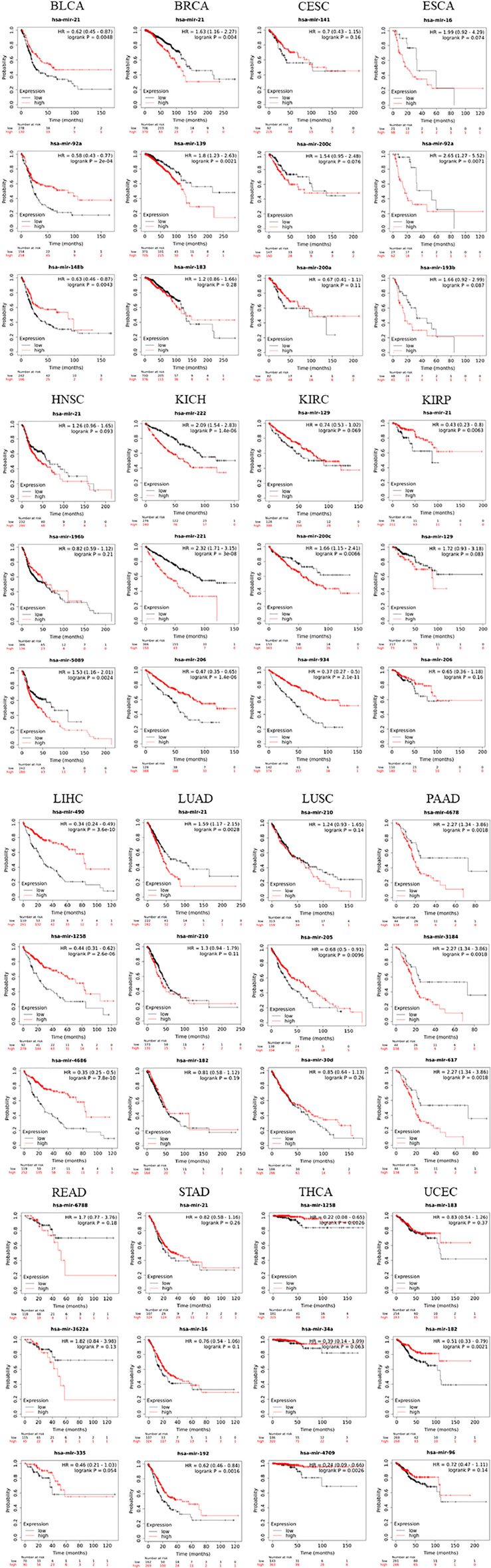
The Kaplan-Meier survival analysis of top-3 ranked miRNAs extracted from the datasets of 20 types of cancer.

The top-3 ranked miRNAs demonstrated drastic variations on the survival of patients (Figure 6), which impacted the prognostic survival of patients in BLCA, BRCA, CESC, ESCA, HNSC, KICH, KIRC, KIRP, LIHC, LUAD, LUSC, PAAD, READ, STAD, THCA and UCEC. Two categories have strongly functioned in a positive or negative manner, respectively. 1) with strong POSITIVE impacts: **miR-21** (HR = 0.43, log_rank *p* = 0.0063 in KIRP; HR = 0.62, log_rank *p* = 0.0048 in BLCA); **miR-92a** (HR = 0.58, log_rank *p* = 2e-04 in BLCA); **miR-148b** (HR = 0.63, log_rank *p* = 0.0043 in BLCA); **miR-182** (HR = 0.51, log_rank *p* = 0.0021 in UCEC); **miR-206** (HR = 0.47, log_rank *p* = 1.4e-06 in KICH); **miR-490** (HR = 0.34, log_rank *p* = 3.6e-10 in LIHC); **miR-934** (HR = 0.37, log_rank *p* = 2.1e-11 in KIRC); **miR-1258** (HR = 0.44, log_rank *p* = 2.6e-06 in LIHC); **miR-4686** (HR = 0.35, log_rank *p* = 7.8e-10 in LIHC); and **miR-4709** (HR = 0.24, log_rank *p* = 0.0026 in THCA). 2) with strong NEGATIVE impacts: **miR-21** (HR = 1.63, log_rank *p* = 0.004 in BRCA; HR = 1.59, log_rank *p* = 0.0028 in LUAD); **miR-92a** (HR = 2.65, log_rank *p* = 0071 in ESCA); **miR-139** (HR = 1.80, log_rank *p* = 0.0021 in BRCA); **miR-200c** (HR = 1.66, log_rank *p* = 0.0066 in KIRC); **miR-221** (HR = 2.32, log_rank *p* = 3e-08 in KICH); **miR-222** (HR = 2.09, log_rank *p* = 1.4e-06 in KICH); **miR-617** (HR = 2.27, log_rank *p* = 0.0018 in PADD); **miR-3184** (HR = 2.27, log_rank *p* = 0.0018 in PADD); **miR-3622a** (HR = 1.82, log_rank *p* = 0.13 in READ); **miR-4678** (HR = 2.27, log_rank *p* = 0.0018 in PADD); and **miR-6788** (HR = 1.70, log_rank *p* = 0.18 in READ). Remarkably, these key miRNAs have been pre-verified by clinical information of patients in the TCGA database and the miRNA-disease association databases (miR2Disease and HMDD); some of them are in line with the accumulated evidences drawn from literature as discussed (in Discussion) later, which enhance their potential of clinical applications. To our knowledge, most of them are uncovered for the first time, thus deserving to be exploited through future experiments.

### Verification of key miRNAs via the Analysis of Sensitivity or Resistance to Anti-Cancer Drugs

**FIGURE 7:**
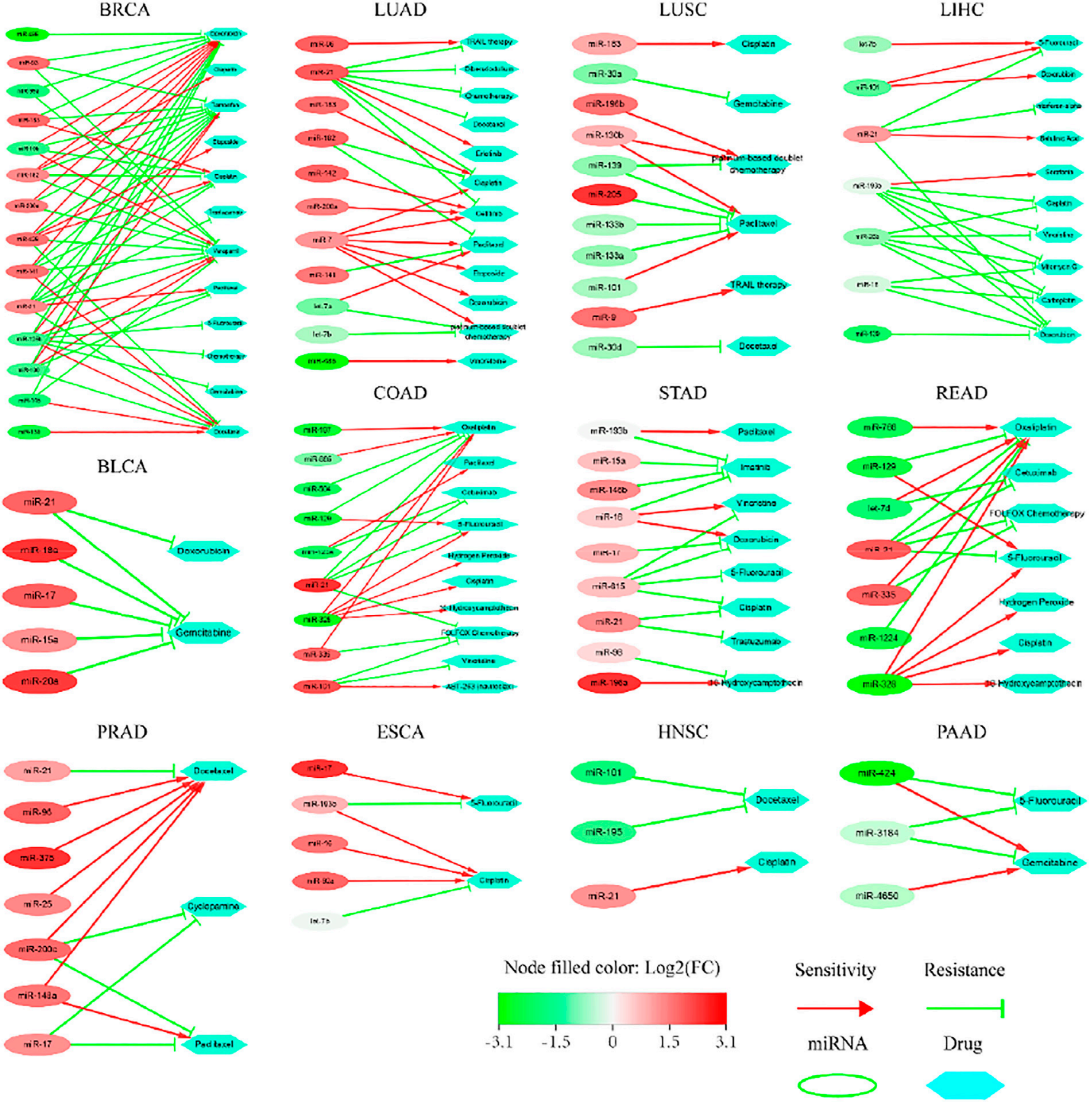
Sensitivity or resistance to anti-cancer drugs by the top-20 ranked miRNAs extracted from the TCGA datasets of 20 types of cancer.

We submitted the top-20 ranked miRNAs to ncDR and mTD, respectively, searching for candidate matches. The results are outlined (Figure 7) below. 1) 14, 9, 11, 7, 11, 7, 7, 9 miRNAs impacted drug resistance or sensitivity in BRCA, COAD, LUAD, LIHC, LUSC, PRAD, READ and STAD, respectively; 2) 5, 5, 3 and 3 miRNAs impacted drug sensitivity or resistance in BLCA, ESCA, HNSC and PAAD, respectively; but 3) none of the miRNAs impacted drug resistance or sensitivity in CESC, CHOL, KICH, KIRC, SKCM, THCA and UCEC. We remind that a possible reason for these fewer matches probably lies in that there are relatively fewer records on these cases in the current version of two databases.

Our data suggest that the abnormal expression of key miRNAs impacted the sensitivity or resistance to anti-cancer drugs; some miRNAs promoted drug sensitivity whereas others increased drug resistance (Figure 7). We highlighted certain cases as follows. 1) One miRNA impacted a number of drugs, which produced different sensitivity or resistance; and vice versa. It was reported that the overexpression of miR-182 in breast cancer caused resistance to Olaparib, Verapamil, Tamoxifen and Cisplatin, but increased sensitivity to Doxorubicin (Kovalchuk et al., 2008). Here, we found more cases. All overexpressed miRNAs in bladder cancer promoted resistance to Gemcitabine. Low expression of miR-129 in colon cancer induced resistance to Oxaliplatin, but increased sensitivity to 5-Fluorouracil. Overexpression of miR-193b in esophageal cancer promoted resistance to 5-Fluorouracil, but increased sensitivity to Cisplatin. Overexpression of miR-200c in prostate cancer promoted sensitivity to Docetaxel, but increased resistance to Cyclopamine and Paclitaxel. Overexpression of miR-7 in lung adenocarcinoma weakened resistance to 6 drugs. Overexpression of miR-130, but low expression of miR-101, promoted sensitivity; while low expression of miR-139, miR-133a, miR-133b, but overexpression of miR-205, increased resistance to Paciltaxel. Overexpression of most miRNAs in gastric cancer was associated with drug sensitivity or resistance. Low expression of most miRNAs in liver cancer was associated with sensitivity or resistance. Low expression of miR-101 and miR-195 increased resistance to Docetaxel, but overexpression of miR-21 promoted sensitivity to Cisplatin in the cancer of head and neck. Low expression of miR-424 in pancreatic cancer promoted sensitivity to Gemcitabine, but increased resistance to 5-Fluorouraci. 2) Strikingly, **miR-21** appeared frequently in multiple datasets. Abnormal expression of miR-21 impacted sensitivity or resistance to multiple drugs in BRCA, BLCA, PRAD, LUAD, STAD, HNSC, LIHC and READ. The mechanisms underlying these candidates remained elusive. Collectively, these key miRNAs have complex impacts on the above anti-cancer drugs, which not only illustrate their potential roles in tumorigenesis, but also provide a new perspective for precision medicine.

### Comparison on the Performance of the MIMRDA Method Over Existing Methods

**FIGURE 8:**
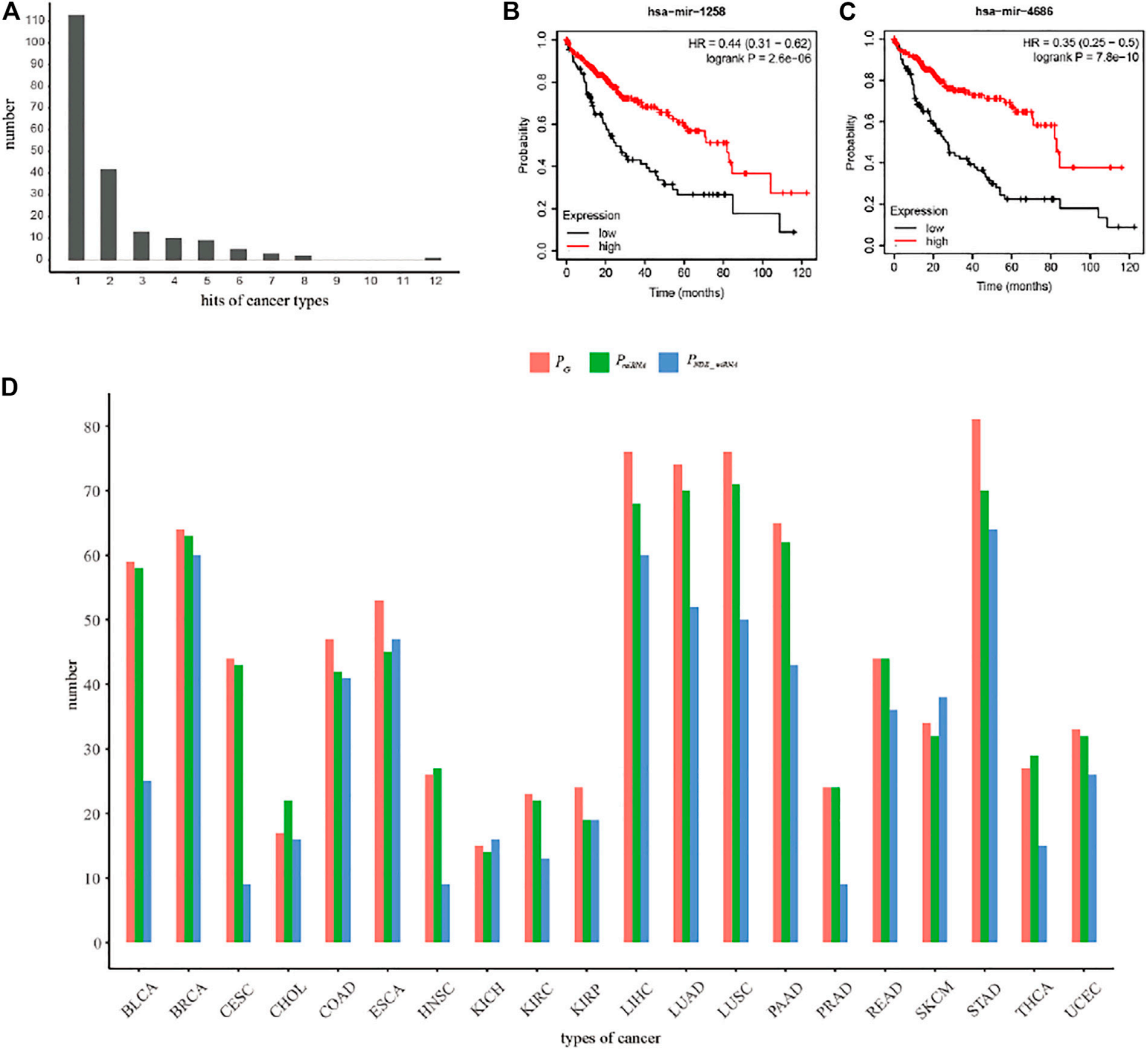
The performance comparison of the MIMRDA method over other methods. **(A)** The number distribution of top-20 ranked cancer-related miRNAs. **(B)** The survival analysis of miR-1258 in LIHC. **(C)** The survival analysis of miR-4686 in LIHC. **(D)** The performance comparison among the MIMRDA method (*P*_*G*_), the Limma package (*P*_*miRNA*_) and the SPIA package (*P*_*NDE_miRNA*_) based on the top-100 ranked miRNAs identified from the TCGA datasets of 20 types of cancer.

To illustrate the superiority of the MIMRDA method, we compared the miRNAs that were identified by the MIMRDA method, the Limma package (Ritchie et al., 2015) and the SPIA package (Tarca et al., 2009), respectively. For simplicity, we focused on the top-100 ranked miRNAs that were extracted from each dataset of each type of cancer (Figure 8). Note that since the classical approaches utilized the known disease-related miRNAs to establish training sets to prioritize miRNAs (Ritchie et al., 2015), it is impossible to use those prioritization methods based on the expression values of genes (or miRNAs), or an overall performance metrics. Hence, we compared the number distribution of candidate miRNAs (i.e., the known disease-related miRNAs). A method performs better if more disease-related miRNAs are found. Obviously, the MIMRDA method identified more miRNAs related to the known types of cancer, which solidifies the superiority of the MIMRDA method to the counterpart methods. Remarkably, as representatives in the second category, who are not matched with the aforementioned two databases, miR-1258 (Figure 8B) and miR-4686 (Figure 8C) have shown perfect survival rates, which warrant future experimental validations.

### Evaluation on the Performance of the MIMRDA Method via the Random Forest Simulation Test

**FIGURE 9:**
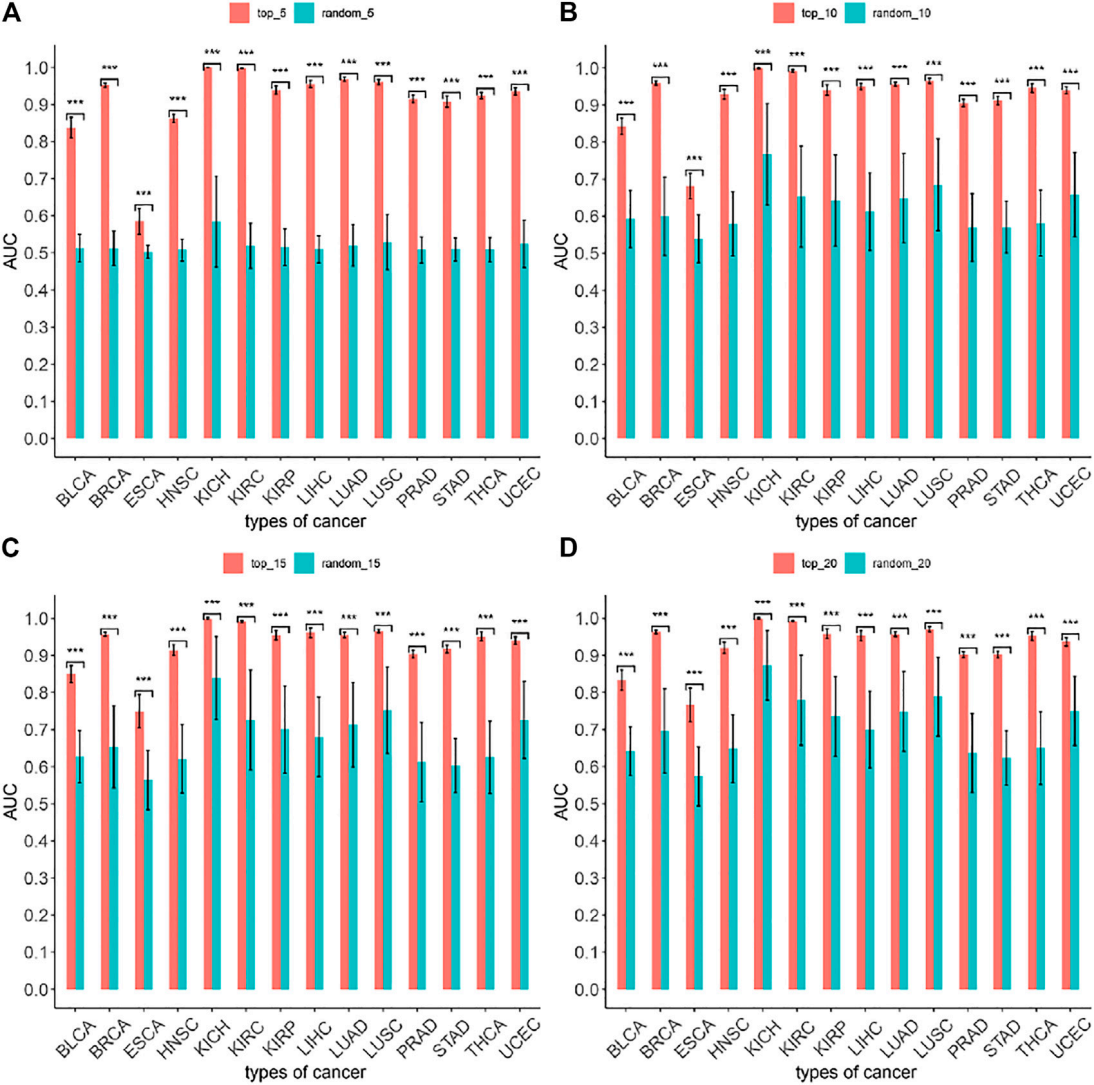
Evaluation on the performance of the MIMRDA method via the Random Forest simulation test. The top-ranked miRNAs identified by the MIMRDA method are compared with the randomly selected miRNAs, both after the five-fold cross-validation Random Forest simulations. **(A)** Top_5 ranked miRNAs *vs* random_5 miRNAs. **(B)** Top_10 ranked miRNAs *vs* random_10 miRNAs. **(C)** Top_15 ranked miRNAs *vs* random_15 miRNAs. **(D)** Top_20 ranked miRNAs *vs* random_20 miRNAs. *p*-value < 0.001***.

The five-fold cross-validation Random Forest simulation test (see Materials and Methods) was applied to evaluate the accuracy of the MIMRDA method in classifying top-ranked miRNAs. The results indicate that the MIMRDA method is significantly (*p*-value < 0.001) better than the random selection in terms of the overall AUC values (Figure 9), suggesting the effectiveness and reliable ability of the MIMRDA method in classifying the top-ranked miRNAs.

## Discussion

The proposed MIMRDA method identified hundreds of top-ranked miRNAs from TCGA datasets of 20 types of cancer, and recommended them warrant further validations. We employed miR2Disease (Jiang, et al., 2009) and HMDD 3.0 (Huang, et al., 2019) to infer the miRNA-disease associations based on the pre-verified evidences. We deployed MISIM 2.0 (Li et al., 2019) to infer the function similarity of key miRNAs based on the pre-verified function similarities. We applied ncDR (Dai et al., 2017) and mTD (Chen et al., 2017a) to infer the sensitivity or resistance to anti-cancer drugs based on the pre-verified miRNAs-drug associations. Such that our findings were cross-verified to one another. We conclude that most of the top-ranked key miRNAs are the cancer-related miRNAs deposited in miRTarBase (Huang et al., 2020) and TCGA (https://portal.gdc.cancer.gov/) databases, while some are supported by literature evidences. We highlight some key miRNAs that are well supported by the accumulated experimental evidences recaptured from literature, thus highlighting their potential to be biomarkers, which should be valuable to the community.

Firstly, the majority of top-ranked miRNAs (as Category 1, e.g., **miR-21**) are endorsed by the pre-verified relationship of miRNAs-cancer in the state-of-the-art databases (Figures 4–7), suggesting that they are truly cancer-related miRNAs and have high potentials to be biomarkers. Here are some examples highlighted with the experimental evidences drawn from literature. 1) **miR-16** inhibited the proliferation and migration of gastric cancer cells by targeting SALL4 (Jiang and Wang 2018). 2) **miR-21** was up-regulated in gastric cancer, and its dysfunction had a critical role in gastric cancer growth and dissemination by regulating PTEN and PDCD4, plus by modulating the pathways involved in mediating cell growth, migration, invasion and apoptosis (Li Y. et al., 2014). **miR-21** and **miR-155** promoted the development of non-small cells by down-regulating SOCS1, SOCS6 and PTEN (Xue et al., 2016). **miR-21** significantly reduced or increased epithelial-mesenchymal transition (Dai et al., 2019). Overexpression of **miR-21** in non-small cell lung cancer up-regulated the expression of cyclin D1 and cyclin E1, respectively (Dai et al., 2019). 3) **miR-34a** was overexpressed and used as a potential target for thyroid cancer (Shabani et al., 2018). 4) **miR-182** targeted CTTN in non-small cell carcinoma to inhibit the formation of aggressive pseudopodia in lung cancer, inhibiting the metastasis of lung cancer (Li et al., 2018). 5) **miR-192-5p** was down-regulated in gastric cancer, as a potential diagnostic target (Tavakolian et al., 2020). 6) **miR-210** promoted the development of lung cancer by targeting LOXL since down-regulation of LOXL4 significantly inhibited the proliferation, migration and invasion of lung cancer cells in A549 and H1650 cell lines (Xie et al., 2019). 7) **miR-335** exhibited a tumor suppressor effect by inhibiting Twsit1 in colorectal cancer (Wang et al., 2017), whereas miR-3065-3p promoted stemness and metastasis by targeting CRLF1 in colorectal cancer (Li et al., 2021). 8) **miR-490-5p** was related to tumor size, tumor metastasis stage and survival rate of HCC patients because miR-490-5p inhibited HCC cell metastasis by regulating E2F2 and ECT2 (Fang et al., 2018). Therefore, such experimental evidences in literature are in line with our findings of some top-ranked key miRNAs.

Secondly, some top-ranked key miRNAs (as Category 2, e.g., **miR-1258**) are not matched with the above databases, but they were well supported by the experimental evidences drawn from literature. For instance, among the top-20 ranked miRNAs, two (**miR-1258** and **miR-4686**) were not matched with miR2Disease and HMDD, respectively, despite that the rest 18 related to LIHC did match. However, we found that miR-1258 and miR-4686 were down-regulated in tumor samples when comparing 375 samples of liver cancer with 50 normal samples (data not shown). We performed the KM survival analysis of miR-1258 and miR-4686 (Figures 7B,C), respectively, based on the miRNA expression profiles in 375 samples of primary liver cancer alongside clinical information from TCGA database, and found the significant (*p*-value < 0.001) survival. Our data suggest that miR-1258 and miR-4686 are likely the potential prognosis factors in LIHC. In fact, miR-1258 was reported significantly down-regulated in liver cancer samples that closely related to the poor survival of patients (Hu et al., 2016), which is consistent with our data. Moreover, loss of miR-1258 led to the initiation and development of liver cancer by targeting CKS1B (Hu et al., 2016); while overexpression of miR-1258 inhibited the growth, proliferation and tumorigenicity of liver cancer cells by increasing G0/G1 cell cycle arrest and promoting cell apoptosis (Hu et al., 2016); and miR-1258 exerted anti-cancer function by targeting TMPRSS4 in thyroid cancer (Wang and Cai 2020). Taken together, our findings coincide with the experimental evidences drawn from literature, and suggest that **miR-1258** has the potential to be developed as an independent prognosis factor in liver cancer.

Thirdly, some top-ranked key miRNAs are related to multiple types of cancer, whereas others are related to a single type of cancer (Figure 4). For instance, miR-16-1, **miR-21**, **miR-93**, miR-141, **miR-183** and **miR-193b** present in 7, 12, 8, 7, 7 and 8 types of cancer, respectively, thus impacting the carcinogenesis of multiple types of cancer. Here are examples highlighted. 1) **miR-21** is related to 12 types of cancer (BLCA, BRCA, CESC, CODA, HNSC, KIRC, KIRP, LIHC, LUAD, PRAD, READ and STAD). In fact, miR-21 was experimentally verified to be highly correlated with cancer initiation and metastasis (Liu H. et al., 2018; Wang et al., 2019). 2) **miR-93** is related to 8 types of cancer (BLCA, CHOL, ESCA, KIRP, LIHC, PRAD, STAD and UCEC). In fact, **miR-93** was reported to be closely associated with lung cancer (Li J.-Q. et al., 2017), prostate cancer (Liu J.-J. et al., 2018) and liver cancer (Xu et al., 2018). 3) **miR-183** is related to 7 types of cancer (BLCA, BRCA, CESC, LUAD, LUSC, PRAD and UCEC). In fact, the abnormal expression of **miR-183** initiated multiple types of cancer (Chen X. et al., 2018; Trinh et al., 2019; Li et al., 2020). 4) **miR-193b** is related to 8 types of cancer (BLCA, CESC, CHOL, ESCA, HNSC, LIHC, LUAD and STAD). In fact, miR-193b was reported to be closely associated with breast cancer (Hulin et al., 2017), liver cancer (Yin et al., 2018) and gastric cancer (Song et al., 2018). Besides, some top-ranked key miRNAs were recaptured in details earlier (see Results) to be uniquely related to a single type of cancer. Taken together, we conclude that some top-ranked key miRNAs are either poly- or mono-valence against multiple types or single type of cancer, respectively.

Finally, the majority of top-ranked key miRNAs are positively or negatively involved in the overall prognostic survival, in the context of specific type of cancer (Figure 6). The mechanisms underlying such survival rates remained elusive, but are partly supported by the accumulated experimental evidences drawn from literature. Here are examples highlighted. 1) Abnormal expression of **miR-16** inhibited cell apoptosis by regulating the expression of RECK and SOX6, promoted cell growth and ultimately led to the occurrence of esophageal cancer (Zhu et al., 2014). 2) **miR-21** regulated cell proliferation and sensitivity to Adriamycin in bladder cancer cells (Tao et al., 2011). Overexpression of **miR-21** was highly correlated with poor prognosis of breast cancer (Yan et al., 2008). Overexpression of **miR-21** in T24 cells promoted cell proliferation and resistance to Adriamycin, and resulted in the up-regulation of BLC2, which prevented the apoptosis of T24 cells induced by Adriamycin, favoring the carcinogenic effect of **miR-21** in bladder cell carcinoma (Tao et al., 2011). **miR-21** and PTEN expression had negative correlation *in vivo* in T24 cells (Tao et al., 2011). Low expression of **miR-21** was correlated with poor prognosis of bladder cancer (Zhang et al., 2015). Overexpression of **miR-21** was highly related to the initiation and development of cancer of head and neck (Arantes et al., 2017). **miR-21** promoted the proliferation and metastasis of breast cancer cells by targeting LZTFL1 (Wang et al., 2019). 3) **miR-92a** might be a target for the clinical diagnosis of bladder cancer. Low expression of **miR-92a** was correlated with the poor prognosis of bladder cancer (Motawi et al., 2016). **miR-92a** inhibited the expression of tumor suppressor CDH1. Overexpression of miR-92a restored the metastatic activity of miR-92a, suggesting that miR-92a promoted the migration of esophageal cancer cells by partly inhibiting CDH1. Patients with up-regulated miR-92a were prone to lymph-node metastasis and had a poor prognosis (Chen et al., 2011). 4) **miR-139-3p** exerted a tumor suppressor effect in breast cancer by targeting RAB1A, and might serve as a potential biomarker for prognosis of breast cancer (Zhang et al., 2019). 5) Overexpression of **miR-141** led to the occurrence of cervical cancer (Gómez-Gómez et al., 2013). 6) The serum **miR-148b** markers might have a clinical value in the diagnosis of bladder cancer (Jiang et al., 2015). 7) **miR-183** was dysregulated in breast cancer, related to the expression of estrogen receptor and HER2/neu receptor (Lowery et al., 2010). 8) **miR-193b**/KRAS was expressed in a stage-dependent manner; KRAS was regarded as a direct target of miR-193b; and the upregulation of miR-193b increased the percentage of apoptosis. miR-193b was a biomarker for the treatment of esophageal cancer (Kang et al., 2019). 9) **miR-196a** and **miR-196b** produced cell-specific responses to target genes and downstream pathways, which significantly impacted the cell proliferation, migration and invasion (Álvarez-Teijeiro et al., 2017). Abnormal expression of **miR-196b** presented in the initiation of head and neck cancer. **miR-196b** was a biomarker for early diagnosis of head and neck cancer. 10) **miR-200a** was down-regulated in cervical cancer (Bozgeyik et al., 2020). **miR-200c** inhibited the metastasis and growth of cervical cancer cells via targeting MAP4K4 (Mei et al., 2018). **miR-200c** controlled cell cycle progression and cell growth by down-regulating the G1-S regulator CDK2, and had anti-cancer impacts in ccRCC (Wang et al., 2015). 11) **miR-206** was one of the most critical tumor suppressor miRNAs in ccRCC, which induced cell cycle arrest and inhibited the proliferation of ccRCC cells via targeting CDK4, CDK9 and CCND1 (Xiao et al., 2016). 12) **miR-221** and **miR-222** discriminated the renal cell carcinoma subtypes and tumor cell (Di Meo et al., 2018). 13) **miR-934** was a diagnostic and prognostic biomarker of clear renal cell carcinoma (Liang et al., 2017). Taken together, we conclude that the candidacy of certain key miRNAs identified in this study are supported by experimental evidences recaptured from literature, which provide informative cues for future validations to develop them to be biomarkers ultimately used for the diagnosis and treatment of multiple types of cancer.

We would like to mention possible limitations of our method. We incorporated the mRNA and miRNA expression profiles from the TCGA datasets to identify key miRNAs (microRNAs), rather than utilized other kinds of ncRNAs datasets, such as lncRNAs (Ou-Yang et al., 2019; Lan et al., 2020; Wu et al., 2021) and circRNAs (Liu et al., 2021). Utilizing lncRNAs and circRNAs will be another possible direction of identifying the cancer-related ncRNAs by integrating complex network-based and machine learning-based methods in the future work.

## Conclusion

We introduced the MIMRDA method, which incorporated the expression profiles of miRNAs and target mRNAs for predicting the miRNA-disease association to identified key miRNAs (microRNAs). As a proof-of-principle study, we deployed the MIMRDA method to analyze 10,499 samples from TCGA datasets of 20 types of cancer, and identified hundreds of key miRNAs. Most of them were significantly related to at least one type of cancer under study, which were supported by the pre-verified miRNA-disease/drug association databases. We indicated the superiority of the MIMRDA method to the Limma and SPIA packages, and the accuracy of the method in classifying top-ranked miRNAs. Our results recommended some top-ranked key miRNAs be experimentally validated as biomarkers in the future.

## Data Availability

The original contributions presented in the study are included in the article/Supplementary Material, further inquiries can be directed to the corresponding authors.
